# Dual ectopy: Unique appearance of ectopic thyroid

**DOI:** 10.1016/j.radcr.2024.01.051

**Published:** 2024-02-17

**Authors:** Liam du Preez, Francis Flaherty, Ragaa Elkabbani

**Affiliations:** a Norwalk Hospital Diagnostic Radiology Residency, 34 Maple St Norwalk, CT 06850 USA; b Danbury Hospital Clinical Pathology Residency, 24 Hospital Ave Danbury, CT 068102 USA

**Keywords:** Thyroid, Ectopic, Anatomy, Radiology, Biopsy, CT

## Abstract

A 67-year-old female underwent a computed tomography angiogram (CTA) of the head in the setting of acute, short-term memory loss. Two lobulated hyperattenuating lesions were incidentally discovered at the base of the tongue and the hyoid bone. Upon further investigation in the outpatient setting including further imaging and ultrasound-guided biopsy, the lesions were confirmed to be ectopic thyroid tissue with dual ectopy. Heterotopic tissue, especially when arising at separate sites, can be concerning for a broad differential diagnosis including malignancy, and further evaluation is certainly recommended. When evaluating possible heterotopic tissue, one must always keep in mind the expected embryologic development of the organ in question. Further, in cases where biopsy is less favorable, consideration of the heterotopic tissue's expected physiology is equally important. With these 2 facts in mind, midline hyperattenuating, enhancing lesions in the neck must always be considered to be a possible developmental anomaly of the thyroid, even when there are multiple lesions.

## Introduction

Heterotopic locations of organs have myriad manifestations, pathophysiology, and clinical significance. With modern imaging techniques, heterotopias can be seen as early as the prenatal period or may go undetected until autopsy. Differential diagnoses for incidentally noted heterotopic tissue can include malignancy (both primary and metastatic), infection, sequelae of prior trauma, or developmental events. When incidentally identified masses occur in specific patterns, synthesis of laboratory results, imaging, pathology, and embryological development can narrow the differential significantly. This case report features a rare presentation of dual ectopic thyroid in a 67-year-old adult with hypothyroidism and exemplifies this process.

Ectopic thyroid tissue is the most common form of thyroid dysgenesis, occurring once in every 100,000-300,000 people [1]. Greater than 75% of cases occur in females, and presentation can be variable. Hypothyroidism is a common association, but many euthyroid patients are incidentally found on imaging [2]. Other reported cases describe dysphagia, dysphonia, and rarely symptoms of airway obstruction [3]. Not only are symptoms and associations broad, but the location of the ectopia is highly variable. Ectopic thyroid tissue is usually found along the pathway of descent of the thyroglossal duct, extending from the foramen cecum to the level of the thyroid cartilage. In extremely rare cases, primitive thyroidal tissue can seed multiple locations, such as in the case of dual thyroid ectopy.

## Case report

**Fig. 1:**
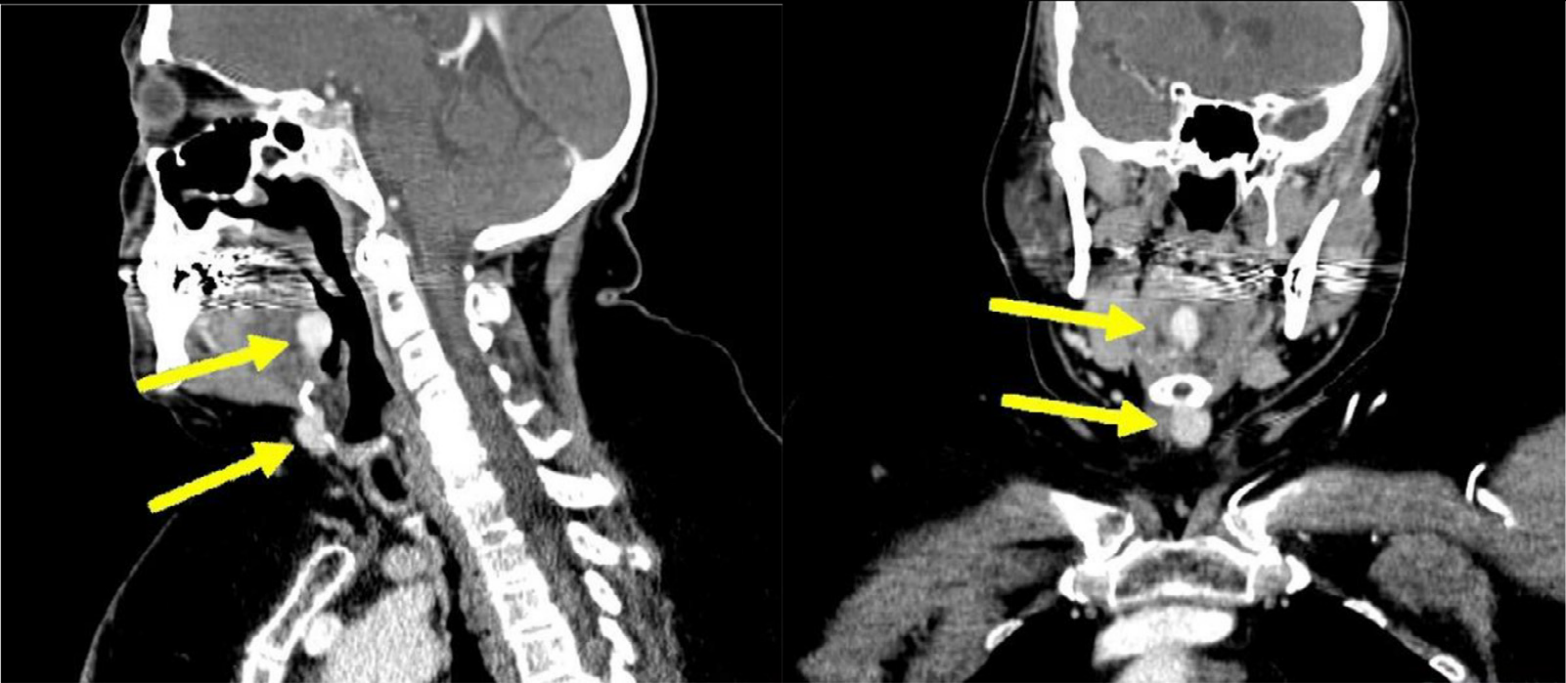
Contrast-enhanced CT of Neck Masses: Sagittal and Coronal images of a contrast-enhanced CT of the neck showing the 2 sites of incidentally noted hyperdense tissue, the more superior of the 2 being at the base of the tongue, the inferior being anterior and adjacent to the hyoid bone.

A 67-year-old female with a significant past medical history of hypothyroidism underwent a computed tomography angiogram (CTA) of the head and neck at an outside hospital for evaluation of cerebrovascular accident in the setting of acute short-term memory loss. While there was no finding of large vessel occlusion, 2 incidental neck masses were identified. Outpatient follow-up with ENT was recommended, and upon further assessment, the patient underwent a contrast-enhanced computed tomography (CT) of the neck. Two similar lesions were identified: a 1.3 × 1.0 × 1.7 cm hyperdense mass of the mid-tongue base and a 1.5 × 1.2 cm hyperdense lesion just inferior to the hyoid bone. The thyroid gland was noted to be absent (Fig. 1).

**Fig. 2:**
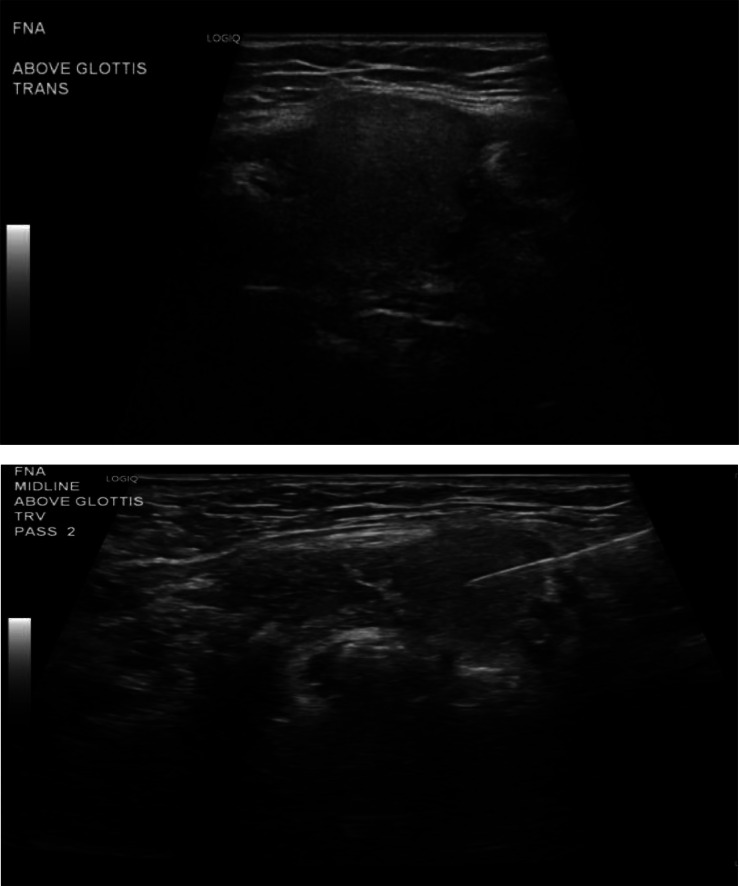
Ultrasound-guided biopsy images: Two intraprocedural images show the hyoid lesion in transverse orientation under ultrasound, with the second image showing percutaneous fine needle aspiration of the same lesion. The tongue-based lesion was sampled as well (not shown).

While the presumptive diagnosis was ectopic thyroid tissue, the differential diagnosis remained broad and included possible malignancy. Thus, ultrasound-guided percutaneous fine needle aspiration (FNA) of both lesions (Fig. 2) was performed by Interventional Radiology. Samples were placed in Cytolyt and Thyroseq suspensions and sent to Pathology.

**Fig. 3.1:**
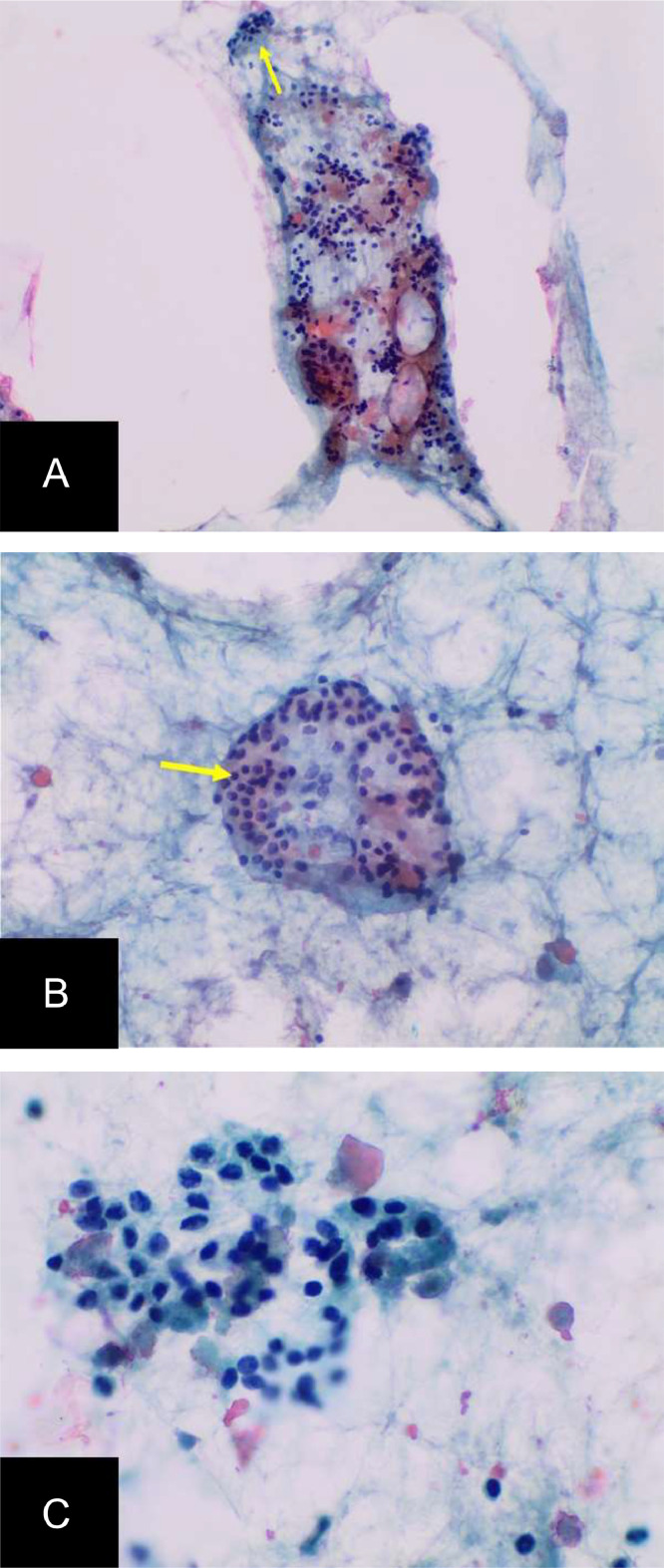
Pathology Images from fine needle aspiration of hyoid bone lesion: Cytologic pictures of direct alcohol-fixed papanicolaou-stained smears show colloid (arrow, a) and clusters of benign follicular epithelial cells (arrow, b) at 20x magnification (A and B), and 40x (C), consistent with benign heterotopic thyroid tissue.

**Fig. 3.2:**
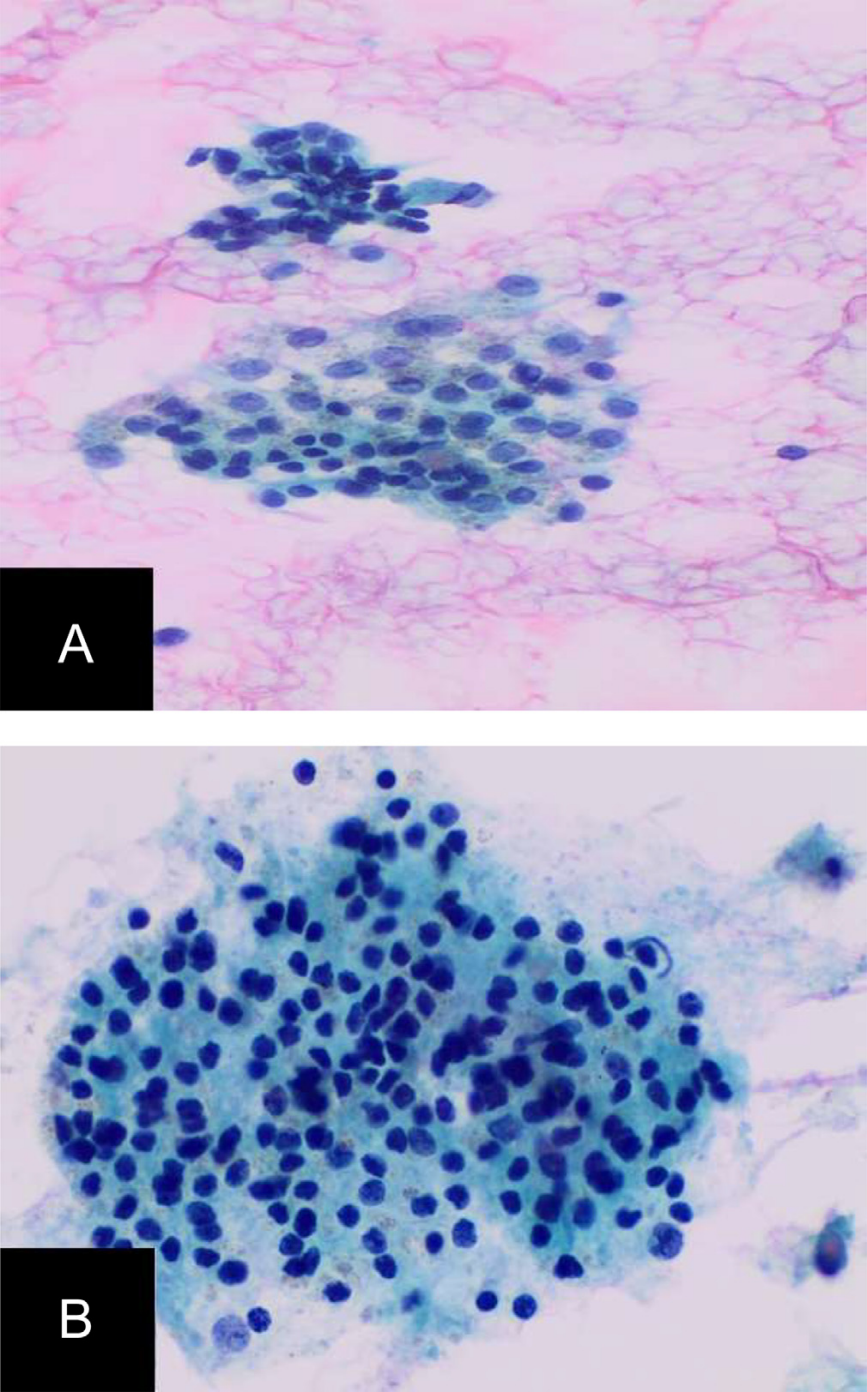
Pathology Images from fine-needle aspiration from base of the tongue lesion: This shows clusters of bland follicular cells and (B) Hürthle cells in a background of thin watery colloid; Direct, alcohol fixed pap-stained smear, magnification 20x magnification (A), ThinPrep CytoLyt Solution, 40x magnification (b).

Samples from both sites showed clusters of benign follicular epithelial cells and colloids, consistent with ectopic thyroid tissue (Fig. 3.1, Fig. 3.2). The patient did not experience any side effects from the procedure and continued with the management of her hypothyroidism without issue.

## Discussion

**Fig. 4:**
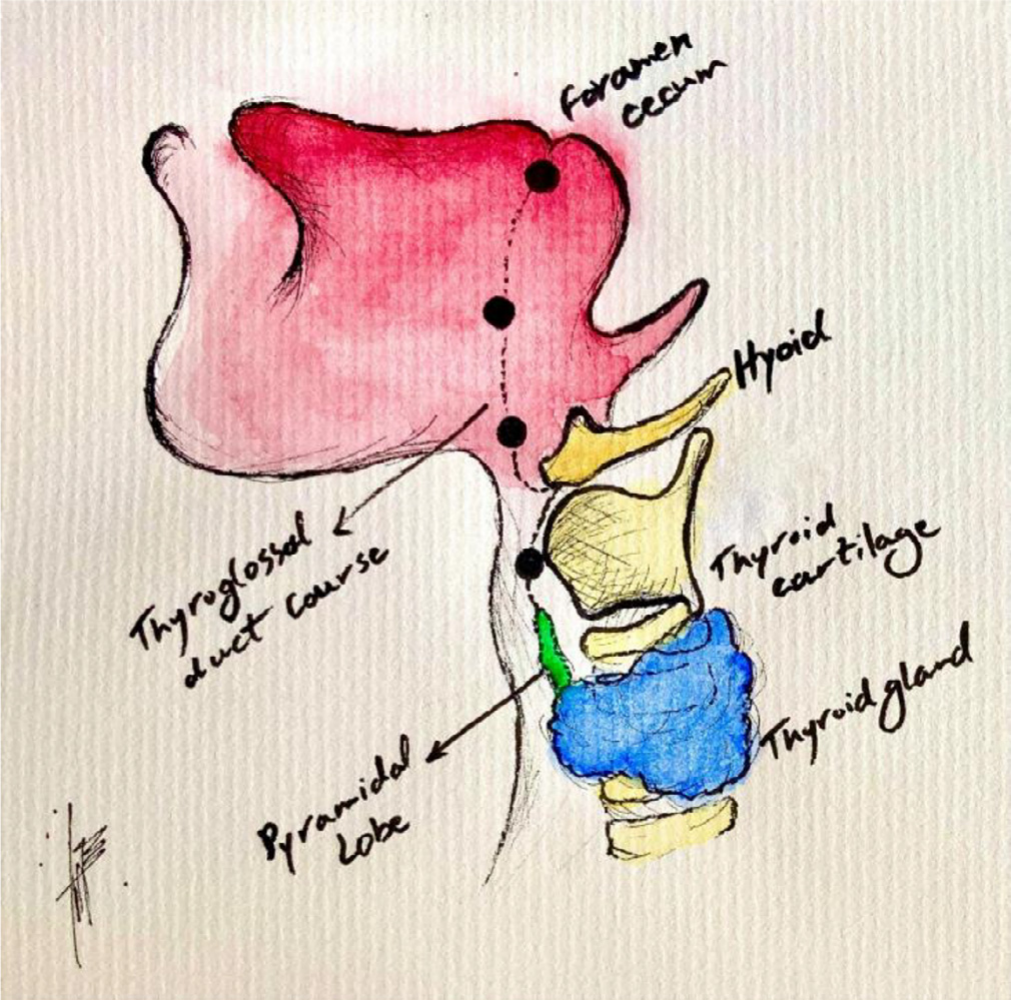
Foramen cecum and thyroglossal duct illustration: illustrated diagram of the thyroid gland's developmental descent via the foramen cecum. Image courtesy of Dr. Behnaz Khazai, MD.

Ectopic thyroid tissue is secondary to incomplete migration of primitive foregut tissue and may appear anywhere along the migration pathway from the foramen cecum to the paratracheal region, as well as rarely in the mediastinum, and subdiaphragmatic regions (Fig. 4). The most common ectopic location is lingual, which appears in approximately 90% of cases [4]. Symptoms manifest broadly and include dysphonia, dysphagia, globus sensation, and airway obstruction. There is also an association with hypothyroidism [5]. Thyroid ectopy has a female predominance and can manifest at any age, but most commonly within the first 3 decades of life.

Ectopic thyroid, as well as various other thyroid abnormalities, has been found to be associated with congenital hypothyroidism [2]. Specifically in cases such as ours with lingual ectopy, hypothyroidism occurs in as much as 70% of patients [3]. The increased attenuation within the lesions on noncontrast CT is secondary to physiologic iodine content. Our case demonstrates a rare occurrence of dual thyroid ectopy, with ectopic thyroid tissue in the tongue base and infra-hyoid neck. Important considerations in the differential diagnosis include lymphoma, metastatic thyroid cancer, neurogenic tumor, and mesenchymal/thymic tumors.

This case was deemed valuable for both imaging and clinical insights into those with heterotopic tissue. For example, while tissue biopsy is certainly the most definitive means of confirming benign thyroid tissue, multiple nuclear medicine scans could also have helped confirm the diagnosis. Thallium-201 especially is useful in not only the detection of thyroid tissue but also in separating malignant from benign based on radiotracer washout characteristics [6]. However, nuclear medicine scans such as this weren't readily available at our institution. Further, in this case, both lesions were amenable to biopsy. In many patients, one or both lesions may not be possible to be biopsied, like cases that involve mediastinum. Again, a nuclear medicine scan would be a less invasive option for securing the diagnosis.

Finally, while this manifestation remains exceedingly rare, dual ectopic thyroid is an important differential diagnosis to consider with multiple homogeneously enhancing neck masses specifically in the pattern of midline distribution along the expected descent of the thyroglossal duct, especially alongside absence of the typical thyroid gland.

## Patient consent

Consent was obtained by the corresponding author and a consent form was completed and scanned into the patient's electronic health record.
